# Blood and Brain Metabolites after Cerebral Ischemia

**DOI:** 10.3390/ijms242417302

**Published:** 2023-12-09

**Authors:** Eva Baranovicova, Dagmar Kalenska, Peter Kaplan, Maria Kovalska, Zuzana Tatarkova, Jan Lehotsky

**Affiliations:** 1 Biomedical Center BioMed, Jessenius Faculty of Medicine, Comenius University in Bratislava, Mala Hora 4, 036 01 Martin, Slovakia; eva.baranovicova@uniba.sk; 2 Department of Anatomy, Jessenius Faculty of Medicine, Comenius University in Bratislava, Mala Hora 4, 036 01 Martin, Slovakia; 3 Department of Medical Biochemistry, Jessenius Faculty of Medicine, Comenius University in Bratislava, Mala Hora 4, 036 01 Martin, Slovakiazuzana.tatarkova@uniba.sk (Z.T.); 4 Department of Histology and Embryology, Jessenius Faculty of Medicine, Comenius University in Bratislava, Mala Hora 4, 036 01 Martin, Slovakia

**Keywords:** cerebral ischemia, animal models, stroke, metabolites, blood, tissues, cerebral microdialysis

## Abstract

The study of an organism’s response to cerebral ischemia at different levels is essential to understanding the mechanism of the injury and protection. A great interest is devoted to finding the links between quantitative metabolic changes and post-ischemic damage. This work aims to summarize the outcomes of the most studied metabolites in brain tissue—lactate, glutamine, GABA (4-aminobutyric acid), glutamate, and NAA (N-acetyl aspartate)—regarding their biological function in physiological conditions and their role after cerebral ischemia/reperfusion. We focused on ischemic damage and post-ischemic recovery in both experimental—including our results—as well as clinical studies. We discuss the role of blood glucose in view of the diverse impact of hyperglycemia, whether experimentally induced, caused by insulin resistance, or developed as a stress response to the cerebral ischemic event. Additionally, based on our and other studies, we analyze and critically discuss post-ischemic alterations in energy metabolites and the elevation of blood ketone bodies observed in the studies on rodents. To complete the schema, we discuss alterations in blood plasma circulating amino acids after cerebral ischemia. So far, no fundamental brain or blood metabolite(s) has been recognized as a relevant biological marker with the feasibility to determine the post-ischemic outcome or extent of ischemic damage. However, studies from our group on rats subjected to protective ischemic preconditioning showed that these animals did not develop post-ischemic hyperglycemia and manifested a decreased metabolic infringement and faster metabolomic recovery. The metabolomic approach is an additional tool for understanding damaging and/or restorative processes within the affected brain region reflected in the blood to uncover the response of the whole organism via interorgan metabolic communications to the stressful cerebral ischemic challenge.

## 1. Introduction

Stroke is ranked as the leading cause of disability and the second leading cause of death worldwide. Four years ago, there were 12.2 million incidents of stroke globally, of which approximately 64% were ischemic strokes [1]. Further data suggest that brain ischemia with recirculation may trigger the pathology of the folding protein characteristic of Alzheimer’s disease through the production and accumulation of amyloids and tau proteins, thus also making it an important factor in triggering the symptoms of Alzheimer’s disease [2,3,4,5,6,7,8]. During cerebral ischemia, brain cells are impacted, and if the blood supply is not restored within minutes, irreversible cell damage and/or death occur as a result of the initial ischemic insult [9]. Ischemic stroke causes a complex series of interconnected events that are not always linear (linear chain of coupled reactions) but often circular (running in a circle), sequential (linked, branched reactions), or causal. The disruption of the oxygen supply to the neuronal cells leads to a shift from aerobic to anaerobic glycolysis, and an overall disordered intraparenchymal metabolism follows the deficiency of energy substrates. The excess of extracellular glutamate due to the disrupted transport of ion and glutamate, in parallel with an imbalance in free radicals, zinc toxicity, as well as alterations to the hydrolytic enzymatic activity, ends in serious neuronal damage. As regulators of ion homeostasis, metabolism, and energy supply, astrocytes undergo reactive gliosis and initiate a series of damage mechanisms during ischemia [10]. They can also play a protective role through the heterogeneous and gradual changes in their gene expression, morphology, and function, working as a double-edged sword. After the restoration of blood flow, the delivery of oxygen and nutrients, such as glucose, is re-established, and potentially damaging by-products of cellular metabolism are removed. Paradoxically, reperfusion causes an exacerbation of ischemic injury by enhancing redox imbalance, a profound inflammation resulting from microglia activation, and subsequent oxidative damage, an occurrence known as reperfusion injury [9].

The complexity of cerebral ischemic/reperfusion damage has been demonstrated in many extensive studies at histological, genomic, transcriptomic, proteomic, and metabolic levels. The latter, a metabolomic approach, was studied only recently by our and other groups. Its advantage lies, for example, in its high sensitivity and reproducibility, the vast knowledge gained from years of biochemical research, and the relatively small number of recognized endogenous metabolites/molecules related to the number of genes, RNA species, or proteins. It is now widely accepted that metabolites are not only intermediates, but also act as important mediators, signaling and regulatory molecules, neurotransmitters, osmolytes, modulators of immunity response, and signalers of epigenetic changes. In addition, single metabolites or their clusters are of high potential in discriminatory analysis in the search for putative disease biomarkers.

So far, metabolomic changes found after cerebral ischemia by us and other groups were summarized in the blood [11,12,13,14,15], in extracellular and cerebrospinal fluid (ECF/CSF) [16,17,18], as well as in the brain [19]. However, little data have been provided and critically discussed to substantiate the veritable physiological role of the most important metabolites in the brain and systemic blood circulation in response to an ischemic injury.

This work is based on our experimental results and also on other studies with the aim of summarizing and discussing the role of metabolite fluctuations involved in cerebral energy metabolism and changes in the level of the most important amino acids. This paper also sums up their biological role in the physiological state compared to ischemic/reperfusion conditions and highlights their role in post-ischemic recovery in both experimental and clinical studies.

## 2. Literature Used in This Work

A search of the literature was performed using the PubMed, Scopus, and Google Scholar databases. We implemented the results from the relevant experimental studies performed by us and other groups as well as human studies focused on metabolic alterations in the brain (metabolites: lactate, glutamate, GABA, glutamine, NAA) and in blood circulation (metabolites involved in energy metabolism and selected amino acids) in relation to cerebral ischemia.

## 3. Stroke in Humans and Animal Models of Cerebral Ischemia

The classification of stroke subtypes, according to Amarenco et al. [20], distinguishes between ischemic stroke, hemorrhagic stroke, subarachnoid hemorrhage, and cerebral venous thrombosis. The most common type in humans is ischemic stroke, which occurs by blocking the blood supply to the affected area of the brain; a hemorrhagic stroke is less common, occurring when an artery in the brain leaks or bursts. The definite cause of stroke remains unclear in almost 25% of cases [20]. Comparison studies on recovery outcomes in ischemic and hemorrhagic strokes yielded mixed results [21,22,23,24,25]. This could be partly explained by the more complex pathophysiology of the certain types of ischemic and hemorrhagic strokes, age, gender, and race [26,27,28]. So far, no literature data provide differences in the metabolomic responses in plasma for both subtypes in clinical studies, likely due to uncertainty and the low discrimination of particular metabolites.

Animal models of cerebral ischemia are characterized as a global, focal, and multifocal restriction of cerebral blood flow, which are mostly carried out on rodents to mimic real human pathology. Global ischemia occurs when the cerebral blood flow is reduced in all regions of the brain, whereas focal ischemia is a reduction in blood flow to a very distinct, specific brain region. In multifocal ischemia, there is an uneven distribution of reduced cerebral blood flow. In complete brain ischemia, cerebral blood flow stops completely, whereas in incomplete ischemia, cerebral blood flow is severely reduced, and the amount of flow is insufficient to maintain cerebral metabolism and function. In focal cerebral ischemia, there may be no blood flow in the core of the ischemia; however, there is usually some flow that reaches the area via collateral circulation [29]. As far as strokes are concerned, two regions are defined as follows: a central core with severely compromised cerebral blood flow (CBF), surrounded by a rim of moderate ischemic tissue with impaired electrical activity but preserved cellular metabolism and viability. This “penumbra” has a variable outcome, and tissue salvage may be achieved when reperfusion is established within a 6 to 8 h period [30]. In other words, penumbra is the term used for the reversibly injured brain tissue around the ischemic core; this region is the main pharmacological target for acute ischemic stroke treatment in humans [31]. The delimitation of the ischemic core is often derived from CBF values with a threshold of 30% (compared to normal tissue); however, this is still under debate [32].

According to the duration of ischemia, the animal ischemia models may induce permanent or transient ischemia, where the latter is followed by blood reperfusion and a re-oxygenation period. The issues of animal models of cerebral ischemia, as an important part of basic neuroscience research, have already been comprehensively summarized and critically discussed in numerous reviews, e.g., [29,33,34,35]. As summed up by Li and Zhang [34], an excellent stroke model should ensure that the repeatability of the model will not be affected by technical difficulties and should be suitable for a variety of small and large animals. It is not surprising that particular differences in metabolic response can be detected in various studies, applied to the high variability of models. Another question concerning metabolomic analyses arises when approximations, such as the induction of hypertension, hyperlipidemia, or hyperhomocysteinemia, increase the translational potential for clinical research in preclinical studies [13,34]; this might, however, challenge the proper reliability of metabolic data and should be critically discussed. Below, we discuss data on the alterations of the best-known and analyzed metabolites and their relations in particular animal ischemic models used by our group and other researchers. In addition to this, we also discuss metabolomic data from human studies linked with ischemic stroke.

## 4. Biochemical Changes in Metabolites in the Brain Detected in Cerebral Ischemic Conditions

### 4.1. Lactate—Proposed Dual Role in Neuronal Death and Survival

Lactate is a natural constituent of brain energy metabolism. Under physiological conditions, it is generated and released by normally oxygenated brain cells [36] and via the lactate shuttle system transported between astrocytes and neurons [37]. The one possible source of glucose as a lactate precursor in the neural tissue is glycogen located exclusively in astrocytes [38,39], which is used as protection against hypoglycemic neural injury [38,40]. However, simple calculations demonstrated that the unevenly distributed glycogen in the brain [41] is not an unequivocally prolonged glucose reservoir and could only fuel brain function for a few minutes in the absence of extra brain plasma glucose [39], and, hypothetically, it is rather intended only for local use [40]. Therefore, the main glucose supply for the brain is the blood that remains, via the blood–brain barrier (BBB). Glucose entry into the brain parenchyma is precisely regulated via glucose transporters (GLUT) localized in the endothelial cells of BBB (GLUT1), then in neurons (GLUT 3), and in astrocytes (GLUT 2, 3, 4). GLUTs are critically involved in sensing glucose concentrations in the blood, promoting central nervous and whole-body glucose regulatory processes [42]. The up-and-down-regulation of GLUTs in particular brain cells, including neurons and astrocytes, responds to the changes in brain energy demand and fluctuations in blood glucose concentrations [42].

**Figure 1:**
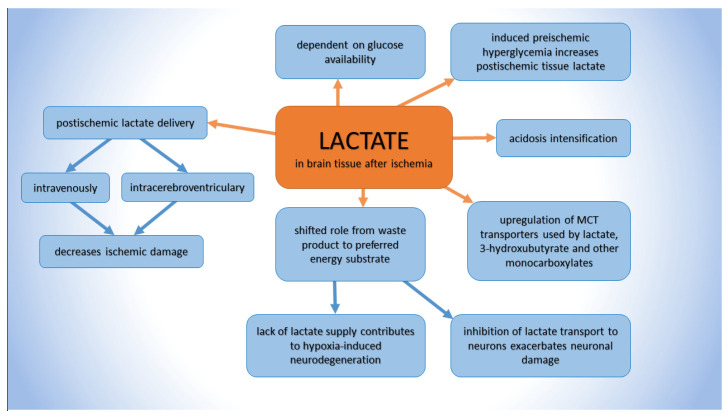
Tissue lactate in relation to cerebral ischemia; MCT—monocarboxylate transporters.

After an ischemic event, the interrupted blood supply to the brain causes a lack of oxygen, anaerobic glycolysis is abruptly accelerated, and lactate production locally increases. The elevated lactate levels in the affected brain region after cerebral ischemia are well documented in animals [43,44], as well as after ischemic stroke in humans [45,46,47,48]. Using ^13^C-labeled glucose, the metabolomic activity of the lactate pool associated with a 32-day-lasting post-stroke event demonstrated that all cerebral lactate arises from the glycolysis of available plasma glucose [49]. Moreover, the post-ischemic production of lactate was found to be dependent on general blood glucose availability. Combs et al. [45] showed that the amount of brain lactate in gerbils after a 20 min ischemic event correlated with the plasma glucose level up to a certain point, where a further increase in blood glucose did not affect the post-ischemic intracerebral lactate level. In a human study, a similar finding was documented when post-stroke brain lactate correlated with the level of blood glucose at patient admission [50]. This is the theoretical substrate for the clinical hypothesis that hyperglycemic conditions before ischemia could lead to an aggravation of post-ischemic brain damage and delayed neuronal damage [51] due to the accumulation of lactate and the intensification of brain intraparenchymal acidosis [52], as was detected in humans [46,53] and in experimental studies [54]. However, this explanation may questioned by several items of data. Many in vitro [55,56,57,58] as well as in vivo studies [59,60,61,62] showed that glucose supply before ischemia was not necessarily harmful and that it could even be beneficial when provided before ischemia [52]. The hypothesis that delayed ischemic neuronal damage is in direct correlation with brain lactate levels (and indirectly with blood glucose levels) was not supported by experiments on hippocampal damage, where, at the same level of post-ischemic lactate, the degree of tissue damage showed remarkable differences [52]. In addition, Schurr et al. [63] presented the conclusion that intracerebrally accumulated lactate was utilized aerobically as the alternative main energy substrate immediately after ischemia/reperfusion. The blockade of lactate transport into neurons, which prevented lactate utilization, consequently exacerbated delayed ischemic neuronal damage [63]. There is also evidence that the lack of lactate supply might strongly contribute to hypoxia-induced neurodegeneration, and a diminished lactate supply from astrocytes could facilitate stroke-induced neurodegeneration [64] (Figure 1).

Further, many studies have proven the fact that lactate has beneficial effects on the recovery from damage caused by an ischemic brain injury, as demonstrated both in animal models [65,66,67,68,69,70] as well as in patients [71,72,73], and lactate’s protective effect was proven in the reperfusion period when administered intracerebroventricularly [66,67] or intravenously into the bloodstream [74]. However, the mechanisms by which the administration of exogenous lactate provides protection are yet to be unraveled. The positive effect of lactate on the outcome of cerebral ischemia does not necessarily contradict its participation in neuronal damage, mainly as a consequence of linearly progressive tissue acidosis corresponding with an increase in lactate during cerebral ischemia [75]. The study by Brouns et al. [76] showed that post-stroke lactate in cerebrospinal fluid, but not in blood, could be useful as a predictor for the clinical outcome (tissue not measured). However, so far, no study has confirmed the direct relationship between the amount of post-ischemically formed brain lactate and the ischemic outcome, which may be concealed in the dual lactate effect.

In addition, a reasonable issue concerns the question of methodology in obtaining real data, namely by the direct lactate measurements in vivo by non-invasive in vivo MRS (magnetic resonance spectroscopy). Chang et al. [77] reported that this method might capture only 25% of the lactate present in the hypoxic or ischemic rat brains. In clinical MR spectroscopy at higher field strengths, lactate might show a reduced or absent signal intensity to hide real conditions. However, this could be avoided by using improved protocols to increase the sensitivity of lactate detection [78]. On the other hand, in vitro methods for determining post-ischemic lactate levels in the brain encounter a methodological problem. Firstly, the moment of tissue removal initiates a spontaneous increase in lactate within seconds, and this phenomenon compromises the determination of the exact value of lactate produced exclusively by the ischemic event.

The fate of post-ischemically increased brain lactate in the reperfusion/reoxygenation period can act as an alternative energy substrate, and enters the tricarboxylic acid cycle to maintain ATP production as efficiently as glucose via pyruvate/acetylCoA. This is probably the reason why only a few studies have documented elevated lactate levels in the bloodstream after a cerebral ischemic event within minutes of ischemia. Rehncrona et al. [79] observed increased whole blood lactate; however, this was analyzed in blood samples in liquid nitrogen, where cell rupture is certain to have played a key role. In patients after a stroke, an elevated lactate level was observed within 72 h in blood plasma [80] and within seven days after the onset of the ischemic event in blood serum [81], but many other experimental studies including ours did not confirm these results [11]. Experimental studies also showed diverse results regarding post-ischemic blood lactate: an increase in 12 h following the middle cerebral artery occlusion (MCAO) model [82], or even a decrease in the model of global cerebral ischemia in rats in 3 h and 24 h after reperfusion in blood plasma [83,84,85,86]. Therefore, the unquestionable increase in tissue lactate after an ischemic injury is not always reflected in plasma. So far, no data exist about the tissue lactate clearance at the reperfusion period using in vivo MRS. Paradoxically, plasma lactate, as the ‘main ischemic metabolite’, was not reproducibly recognized in clinical studies as a metabolite promising any predictive value in the estimations of the outcome after a cerebral ischemic event.

### 4.2. Glutamate, Glutamine, and GABA in the Brain in Relation to Ischemia/Reperfusion Brain Damage

A majority of the energy in the brain is released by aerobic glycolysis and the subsequent oxidation of products; its absence causes a depletion in ATP within only a few minutes. In the acute phase, the lack of ATP hampers the physiological function of ion pumps as the cells fail to maintain an ionic gradient [87], leading to excessive glutamate release from neurons as well as glial, acting as a driving force for ischemia-induced excitotoxicity [87,88,89]. The increased extracellular glutamate levels after cerebral ischemia were observed in the early works on rabbit hippocampi [90,91,92,93]. Glutamate is the most abundant free amino acid in the central nervous system (CNS), acting as an excitatory neurotransmitter, and although a large portion of glutamate in the body originates from the diet, it is unable to cross the blood–brain barrier and must be generated in resident CNS cells such as glia. Therefore, measuring glutamate and another amino acids, particularly behind the BBB, i.e., directly in the extracellular fluid of the brain tissue, requires a deeper and critical understanding of the clinical post-stroke pathophysiology [16,17,53,94]. The high levels of parenchymal glutamate in the extracellular space are well established to appear rapidly after the onset of ischemia, and, therewith, increased extracellular glutamate may serve as a qualitative biomarker of a cerebral ischemic event [17]. However, an unambiguous quantitative link between the enhanced release of glutamate, neuronal injury, post-stroke impairment, and disabilities has not been fully established [95]. The trials to eliminate ischemic damage with glutamate receptor antagonists were promising in animal studies, but low efficacy or serious side effects limited their clinical application in humans. Moreover, a significant delay from the onset of ischemia to the time of drug administration may be one of the key reasons for the failure of glutamate receptor antagonists in clinical trials [96,97]. In addition, many preclinical studies revealed decreased glutamate whole tissue content after ischemia [85,86,98,99,100,101]. In humans, a recent preliminary study by Nicolo et al. [102] showed a decrease in glutamate levels on the ischemic brain side compared to the non-ischemic brain side 2–13 days after a stroke using in vivo NMR spectroscopy at 7T without a correlation with post-stroke impairment.

**Figure 2:**
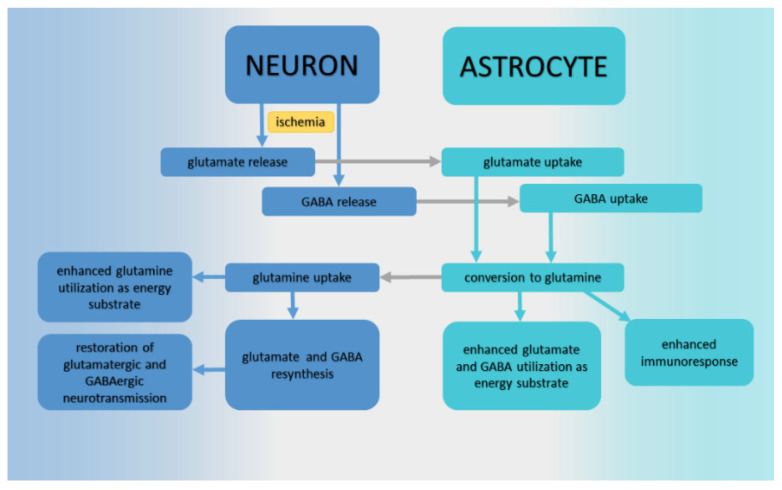
Glutamate, glutamine, and GABA (4-aminobutyric acid) cycles and recovery after ischemia.

In intact conditions, glutamate is localized in synaptic vesicles in neurons, after being released into extracellular space and fulfilling its role as a neurotransmitter. Through its re-uptake in astrocytes, it is metabolized into glutamine by the glutamate/glutamine cycle. Studies on rats showed that brain glutamine synthetase, localized in all cells of the astroglial family [103], increased following cerebral ischemia [104]. Increasing astrocytic glutamine synthetase capacity could, in partial hypoxic/ischemic conditions, improve astrocytes’ ability to convert intracellular glutamate into glutamine, thereby promoting protective glutamate uptake [105]. The other published data, in line with this, showed a massive increase in glutamine tissue content after an ischemic event [85,86], which led to the explanation that post-ischemic glutamate released by neurons began to be immediately converted into non-excitotoxic glutamine by astrocytes (Figure 2). In fact, in our time-related study on ischemic rats’ hippocampi, the massively increased glutamine tissue content after ischemia decreased with a timeline of 3 h, 24 h, and up to 72 h, where it almost reached the level of non-ischemic sham-operated rats [86]. In parallel with this, three days after ischemia, glutamate levels still showed an approximate 10% decrease, signaling that its full replenishment was not achieved. Unfortunately, the studies on tissues using in vitro metabolomic methods cannot easily discriminate the distribution of glutamine among neurons and glia, and verify whether it is still present in astrocytes or already placed in neurons for neurotransmitter re-synthesis.

The overproduction of glutamine in astrocytes, in addition to the protection of neurons from the neurotoxic effects of glutamate or metabolically produced ammonia, might have some metabolic implications for the post-ischemic immunological response. Microglia and astrocytes are the key regulators of neuroinflammation [106], which contribute significantly to the development of ischemic pathology [107]. The inflammatory response is metabolically dependent on glutamine availability, as glutamine serves as the primary fuel for immune cells, microglia, lymphocytes, neutrophils, and macrophages and also appears to play an important metabolic role in cytokine production [108].

It was observed that throughout the course of cerebral ischemia/reperfusion (IR), the synthesis, release, metabolisms, receptors, and transmission of GABA underwent complex pathological variations [109], making GABA a notable participant in IR injury. GABA is an inhibitory neurotransmitter; it can be retaken up by neurons or surrounding glial cells. This is the energy-dependent ion-based process which works against a concentration gradient [110]. GABA in the glia can act as a metabolic substrate and be finally re-converted to glutamine, transferred to neurons, and converted to glutamate, which re-enters the GABA shunt (Figure 2). After a cerebral ischemic event, a massive release of GABA into extracellular space by microdialysis was documented by Zeng et al. [111]. Although, besides microdialysis, the measurement of absolute or relative GABA concentration after ischemia was possible using non-invasive in vivo NMR [112]; surprisingly, only a few studies have been focused on post-ischemic GABA alterations and the dynamics of this process. After a transient ischemic attack, decreased GABA levels were observed in the symptomatic hemisphere [113], and decreased long-lasting GABA tissue content was observed in humans 3 and 12 months post-stroke [114]. Some further studies on brain tissues in vitro documented a decrease in GABA content in ischemic brain parenchyma [85,86,101].

Remarkably, in our study in rats following a cerebral ischemia, we observed a massive decrease in hippocampal GABA content in 3 h reperfusion which gradually increased in 24 h but did not fully recover in 72 h reperfusion [86]. These observations indicate the limited feasibility of a full re-establishment of the proposed GABAergic transmission within a few days of an ischemic event, which is in line with the studies documenting reduced GABA transmission compared with increased glutamate signals [109]: the inhibitory post-synaptic potentials induced by GABA disappeared ex vivo [115], or were weakened in vitro [116] in a short period after ischemia. It appears that a loss of GABA neurotransmission early during reperfusion contributes to ongoing neuronal excitability and excitotoxicity and possibly to neuronal death [117]. A potential preventive strategy for cerebral IR injury is enhancing GABA actions through different enhancers of GABA transmission that could partly restore the balance in GABA and glutamate transmissions [109].

No direct relationship between the amount of glutamate, glutamine, and GABA and the post-ischemic clinical outcome was documented in humans [16]. However, differences in the metabolic response to ischemia have been observed in a preclinical study dealing with ischemic preconditioning, a phenomenon in which tissue is rendered partially or fully resistant to the deleterious effects of the ischemic event by previous exposure to brief periods of hypoxia/ischemia [118]. As we documented in our metabolic study, animals with particular protection manifested a general decrease in neuropathological signs of ischemic injury, showed a lower extent of changes in levels of glutamate, GABA, and glutamine, and a faster metabolic recovery comparable to non-protected animals [86]. These results seem to be very important in terms of a closer correlation between the (i) decrease in pathological ischemic damage, (ii) induction of neuroprotection, and (iii) reduced manifestation of post-ischemic metabolic changes as a new tissue indicator.

In addition to complex damaging glutamate excitotoxic mechanisms, excitotoxic glutamate inhibits mTOR signaling and causes elevated neuronal insulin resistance [119], making neurons more vulnerable to the metabolic stress induced by the ischemic event. On the other hand, this would be a sign of a switch in tissue metabolic demand to using alternative energy substrates as a tool for preserving cell viability. Under physiological conditions, the factual use of glutamate and GABA by astrocytes and glutamine by neurons as useful and real energy substrates is not significant. However, after ischemia, when glucose oxidative metabolism in the brain was decreased, an increase in the metabolic conversion of these amino acids into alternative substrates was observed [120]. It seems that cells with an oxygen restriction might prefer or re-use molecules working as alternative energy substrates and also molecules with other primary functions. The ketone bodies traditionally believed to be an alternative energy supply for neurons are discussed below.

### 4.3. Neuronal Damage via N-Acetyl Aspartate

NAA is an amino acid derivative and the second most abundant metabolite in the brain [121]. To date, there is no generally accepted physiological (primary) role for NAA. It may serve as a reservoir of glutamate [122], a storage substance for acetyl-CoA and aspartate, or a source of the acetyl group for lipid synthesis [123]. Thus, the indirect estimation of brain parenchyma neuronal damage is feasible by the detection of NAA levels due to its almost exclusive localization in neurons [124]. After transient ischemia and brain injury without neuronal death as well as after long-term focal cerebral ischemia, a recovery of altered NAA levels was detected [125], suggesting the position of NAA as a marker of neuronal functionality, rather than of neuronal density [126]. A post-ischemic decrease in NAA might be reversible since the NAA levels in rats subjected to permanent focal ischemia were dramatically reduced in the core infarct at 3 days but partially recovered after 8 days and returned almost to the initial levels by 30 days post-ischemia [127]. Demougeon et al. [125] explained the post-ischemic decrease in NAA levels by the contribution of both the non-functional neurons, expected to contain no or a very low amount of NAA due to fatal injury or death, and of the reversibly injured dysfunctional neurons probably containing less than the normal NAA due to a particular combination of less synthesis and/or a greater release. As NAA is one of the characteristic molecules detectable by fundamental protocols using in vivo NMR spectroscopy (MRS) [47], numerous studies have been conducted on NAA levels in ischemic injury where a typical chronological change has been identified [128], and where an attenuation of NAA levels became evident within an hour of reperfusion and continued with an almost linear decrease during the first 24 h [128].

**Figure 3:**
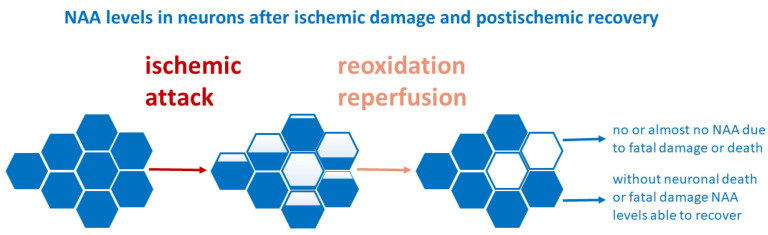
NAA (N-acetyl aspartate) levels in neurons after ischemia and in the reperfusion period: blue filling as visualization of NAA level, as described in text in detail.

In the recent study by our group, the NAA levels in the hippocampus after the utilization of the four-vessel occlusion model in rats decreased in 3 h during reperfusion and further decreased in 24 h, persisting at these levels at 72 h during reperfusion [86]. As pathological/immunochemical features of neuronal damage are easily recognizable only later than 24–48 h after the animal ischemic event, it seems that the determination of NAA levels could be a good opportunity for monitoring incipient or very early neural damage. The post-ischemic alterations in NAA levels seemed to be progressing relatively slowly in comparison to other metabolites, such as glutamate or glutamine [86]. This result is in line with the known phenomenon of delayed neuronal death after ischemia, observable mainly in the hippocampal CA1 region [129]. As experimentally proved, the different tempos together with the possibility that neuronal damage/loss may start in specific brain regions with different intensities. Further, the fact that the resulting decrease in NAA is a combination of both contributing factors—fatally injured neurons and neurons able to recover (Figure 3)—could explain a broad deviation in NAA levels among animals in preclinical studies [85,86]. This inhomogeneity in NAA content might also signalize that the degeneration or viability fate of all neurons in the particular reperfusion time has not yet been decided, and the definite and/or final neuronal injury after ischemia cannot be directly derived or correlated from NAA levels.

## 5. Metabolic Changes Detected in Circulation after Cerebral Ischemia

### 5.1. Role of Blood Glucose in Human Stroke and Preclinical Models

Many studies have been carried out on the analysis and possible impact of blood glucose levels before and/or after ischemia on the severity of cerebral ischemia injury and the tempo of recovery [130,131,132]. It is a well-known fact that hyperglycemia in humans confers a greater risk of a stroke occurring [133]. In contrast, animal studies showed a positive effect of induced hyperglycemia before ischemia on post-ischemic recovery, as already mentioned above [52]. However, there should be a strict distinction between pathological hyperglycemia caused by stress hormones or insulin resistance, which is the failure of tissues to physiologically respond to insulin stimulation and the consequent restriction of glucose transport into the cells [134], and externally induced hyperglycemia, which is evoked exogenously by the intravenous application of dextrose or other sugar solutions before ischemia in experiments.

It was suggested that post-ischemic increased brain lactate is much more elevated in subjects with diabetes mellitus, where coincident hyperglycemia enhances the contribution to ischemic damage; however, literature data are very sparse, and no data can be found to directly confirm this. There are two facts to be considered: Firstly, the amount of post-ischemic increased lactate has not been shown to correlate closely with ischemic outcome, and the addition of exogenous lactate mitigated the detrimental consequences of the cerebral ischemic event. The second fact is that the brain metabolism behaves differently under conditions of insulin resistance. Therefore, the extent of lactate production might not automatically reflect hyperglycemia conditions if it was induced in animals with a physiologically regulated function of insulin, glucose transporters, and metabolic enzymes; moreover, the post-ischemic increased the brain lactate level and was correlated with glucose blood concentrations. Apart from this, it is a well-known fact that diabetes worsens the damage following cerebral ischemia through many other mechanisms, including micro-(involving small vessels, such as capillaries) and macro-vascular (involving large vessels, such as arteries and veins) complications, for example, mostly due to the chronic advanced glycated mechanisms [135].

Post-ischemic increased blood glucose levels may be found in diabetic as well as in non-diabetic patients, where post-ischemic hyperglycemia was recognized as an unfavorable factor related to 3 months of mortality independent of diabetic status [136]. Further, acute post-ischemic hyperglycemia predicted the risk of mortality and poor functional recovery [137], and persistent hyperglycemia after a stroke was correlated with higher 30-day mortality [138]. Changes in glycemia after a stroke in humans cannot be generalized, as different patterns such as persisting normoglycemia, transient, persisting, and delayed hyperglycemia were observed [139]. Stress-induced hyperglycemia, activated after an ischemic event, was not necessarily equivalent to diabetes mellitus-related hyperglycemia, although temporary relative insulin deficiency and an increased rate of hepatic gluconeogenesis count as some of the major factors leading to this condition [140]. Here, systematic and comprehensive studies and a better metabolic and physiological characterization would help understand the acute and delayed response of an organism to an ischemic challenge.

As we have seen in a series of studies in our laboratory, increased blood glucose after severe cerebral ischemia via four-vessel occlusion was documented in rodents [83,84,86] and was manifested as a significant response to severe injury. Remarkably, it did not develop immediately after the ischemic event, and increased blood glucose levels became visible not earlier than after 24 h of reperfusion [84,86,141]. Interestingly, animals in the same studies that underwent ischemic preconditioning, a procedure inducing the particular tolerance to ischemic injury [118], did not exhibit hyperglycemia at the time of reperfusion [84,86]. This finding seems to show a link between developed acute stress hyperglycemia after severe ischemic injury on one side and reduced post-ischemic outcome damage after the preconditioning maneuver, which is associated with ischemic protection on the other side.

### 5.2. Post-Ischemic Changes in Energy Metabolites

Studies on animal ischemia/reperfusion from our group suggest that cerebral ischemia in rats seems to be linked to a generally compromised glucose metabolism, as the levels of glycolytic glucose products (pyruvate, lactate) in the circulating blood plasma were observed to be significantly reduced [83,84,86]. However, in the oxygenated reperfusion period, alternative substrates such as ketone bodies were able to support the metabolic requirements of the brain tissue. In accordance with this, after cerebral ischemia, an increased level of ketone bodies in the blood plasma was observed [83,84,86]. During physiological conditions, ketone bodies can provide as much as 70% of the brain’s energy needs, energetically more efficiently than glucose [142]. It was documented that at the time of acute brain injury, the cerebral uptake of ketones increased significantly [142]. Cerebral ketone uptake also stimulates the increase in cerebral blood flow, and the immediate oxidation of ketone bodies induces a decrease in cerebral glucose uptake despite an adequate glucose supply to the brain [143]. Suzuki et al.’s [144] results indicated that beta-hydroxybutyrate, as the main ketone, was utilized as an energy substrate and might ameliorate the disruption of cerebral energy metabolism after hypoxia, anoxia, and ischemia, where the anaerobic glycolytic pathway was activated. Many previous studies showed that 3-hydroxybutyrate could protect neurons against glutamate-mediated apoptosis and necrosis through the attenuation of reactive oxidant species production [145]. In addition, ketone bodies enhanced the conversion of glutamate to GABA with the subsequent enhancement of GABA-mediated inhibition [146,147], which might help to restore post-ischemic lowered GABA levels (and its neural activity). Interestingly, the lactate levels increased after ischemia enhanced the upregulation of monocarboxylate transporters (MCTs) which transport not only lactate [148] but also 3-hydroxybutyrate [149] and other monocarboxylates [149]. There is a plausible question as to whether the post-ischemic upregulation of MCT transporters with lactate can substantially stimulate the entry of 3-hydroxybutyrate into the neuronal cells after cerebral ischemia.

Unlike animal studies [83,84,85,86,141], metabolomic studies in humans following a stroke did not report a generalized increase in plasma 3-hydroxybutyrate [11,12,13,14,15]; however, the ketone bodies were often not covered by the spectrum of evaluated metabolites in most studies. A plausible explanation for the above-mentioned facts could be suggested as follows: the hyperglycemic condition before and after ischemia, potentially related to less favorable outcomes after cerebral ischemia as a result of previously released stress hormones, can accelerate 3-hydroxybutyrate synthesis in the remote liver, a molecule also easily metabolized in the brain and known for its neuroprotective effects. As shown in our own study on rats, ketogenesis was also metabolically activated in normoglycemia, where a more favorable outcome was expected (however, to a lower extent) [86]. Therefore, the accelerated production of ketone bodies in the extra-cerebrally localized liver after a cerebral ischemic event could be a metabolic alternative option and possibly important in the process of recovery from ischemic brain damage.

It was shown that prophylactic ketosis induced before ischemia improved brain tolerance to ischemia [150,151,152,153], and the increased reliance on ketone bodies appeared to be a form of cerebral metabolic alternation or adaptation [154]. A post-ischemic keto diet could also provide benefits for the treatment of ischemic brain injuries; however, in order to play a neuroprotective role, 3-hydroxybutyrate had to enter the cerebral circulation within a short period [155]. So far, no systematic study has been found to describe the effect of a post-stroke keto diet on outcomes in humans, which would be difficult to introduce in the acute phase, and the comparison among subjects would be suboptimal. However, the exogenous delivery of 3-hydroxybutyrate after an ischemic insult in animals showed expected protection against ischemic brain injury [144,156,157,158,159], revealing that the benefits from increased 3-hydroxybutyrate levels after ischemia seemed to be independent of their origin.

The precise mechanisms whereby (i) caloric restriction, (ii) the ketogenic diet, and/or (iii) ketone bodies themselves might act in the process of ischemic stroke protection are not clear. However, these maneuvers can induce a notable improvement in cellular mitochondrial function, a decrease in tissue inflammatory response, and an increase in the expression of neurotrophins, such as the brain-derived neurotrophic factor [154,160]. The related study by Ciobanu et al. [161] also showed that the behavioral recovery from stroke is enhanced in aged rats by a dietary regimen that reduces body weight prior to a cerebral infarct. Caloric restrictions and a ketogenic diet might be cost-effective and efficient strategies through which stroke incidence, its severity, and/or subsequent pathology could be reduced and could eventually lead to a better recovery [162]. It is noteworthy that the 3-hydroxybutyrate is not only an intermediate metabolite and energy source, but also an important regulatory molecule that could influence gene expression, neuronal function, and the overall metabolic rate [163]. In addition, it could contribute to further epigenetic vascular and intraneuronal changes, such as histone acetylation [164] and DNA methylation [165], to ensure tissue protection and/or partial or full recovery of tissue function and viability.

When dealing with energy metabolism in relation to ischemia, there is a question if the detailed metabolic studies could be useful in the description of the phenomenon known as ‘obesity paradox’, which is the finding of a lower mortality rate on cerebral vascular accidents for people who are overweight or obese within certain subpopulations. Although obesity is described as an undoubted risk factor for developing type II diabetes mellitus (T2DM), it was observed that up to 20 percent of patients with a normal body mass index (BMI), and those with obese BMIs, never develop T2DM [166]. Blood metabolomics can provide an insight into the phenotyping of obesity defined by the BMI and reveal inter-population variability. Individuals carrying obesity-related metabolites have a higher risk of developing various diseases compared with healthy non-obese individuals [167]. Based on metabolic phenotyping, the individuals may be ‘healthily obese’ and ‘unhealthily lean’ phenotypes, which highlights the significance of obesity-related metabolic intricacies for predictive and preventive medicine [166,167].

### 5.3. Post-Ischemic Changes in Circulating Amino Acids in the Liquid Blood Component

Our studies on rat global brain ischemia documented that the condition of elevated glucose in plasma after cerebral ischemia was connected to a simultaneous increase in the plasma levels of branched-chain amino acids (BCAAs) and their corresponding keto forms (BCKAs) [84,86], which are known to be closely linked to glucose and fatty acid metabolism [168]. BCAAs were often elevated in hyperglycemia [168], and it was widely demonstrated that BCAAs upregulated glucose transporters and activated insulin secretion [169], although the mechanism responsible for the increased BCAAs in these insulin-resistant hyperglycemic conditions is not completely clear. Interestingly, in rats subjected to ischemic preconditioning before an ischemic insult displaying not hyper- but normoglycemia, an increase in plasma levels of BCAAs was not observed [86].

**Figure 4:**
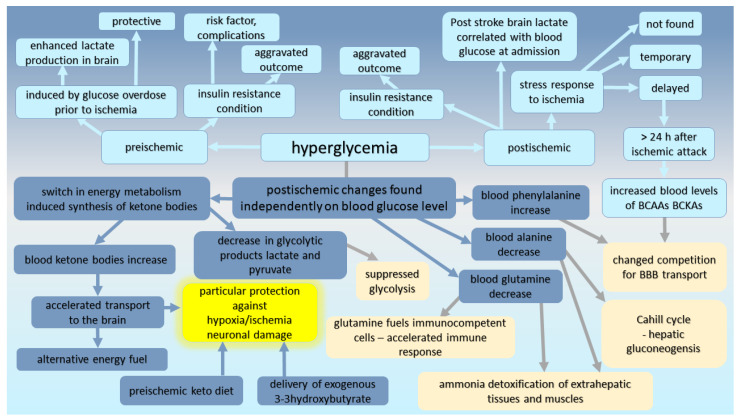
Metabolomic alterations in blood circulation in relation to cerebral ischemia, (BCAAs—branched-chain amino acids, BCKAs—branched-chain ketoacids, BBB-blood–brain barrier).

Not only a hyperglycemic but also a normoglycemic condition after ischemia was accompanied by a decrease in the other groups of important metabolites (Figure 4), all representative of putative gluconeogenetic intermediates, such as pyruvate, lactate, as well as amino acids alanine and glutamine [84,86]. As a result, post-ischemic changes of Cahill (glucose/alanine) and Cori (glucose/lactate) cycle metabolites can affect the well-known link between energy production in the liver and energy production in the muscles. Alanine and glutamine are responsible for the detoxification of metabolically produced ammonia in extrahepatic tissues and muscles (alanine), and their decreased plasma levels suggest a slowdown in the nitrogen shuttle into the liver. In other words, they cause a slowdown of the metabolic rate of nitrogen in an organism. Apart from this, lowered blood plasma glutamine levels, found after cerebral ischemia in animal as well as in human studies [13,80,84,86], might alter the metabolic support needed for the proliferation and function of fast-dividing cells, including immunocompetent cells, for which glutamine serves as an essential energy substrate.

Changes in blood plasma levels of phenylalanine and tyrosine were observed in post-ischemic rats by our group [84] and Gao et al. [170], as well as after a stroke in humans [171] (Table 1). These aromatic amino acids together with tryptophan share common BBB transporters with BCAAs [172,173], and the altered abundances of neutral amino acids in the blood influence their concentration in relation to BBB crossing into the brain. Interestingly, increased brain glutamine, as observed after ischemia in brain tissues [86], also stimulated neutral amino acid uptake by the brain, leading to higher brain concentrations of phenylalanine, tyrosine, and tryptophan than would be expected from amino acid levels (concentration) in the blood [172]. This has an impact on the availability of the substrates for the synthesis of tyrosine-derived neurotransmitters in the brain—dopamine, norepinephrine, epinephrine, and serotonin—affecting individuals’ behavior and brain function, which could play a role in the development of the most frequent neuropsychiatric post-stroke complications, such as post-stroke depression [174] and fatigue [175]. However, the direct measurement of these amino acids in the brain using cerebral microdialysis in humans [17] and animals [176] may shed more light on the distinct alterations of amino acids after a stroke, but also provide a far more complex picture of the pathophysiologic sequela. An increase in phenylalanine was observed after ischemia in rat hippocampi [86], and since it is an essential amino acid, its elevated abundance originated (very likely) only from the blood. Phenylalanine selectively depresses currents at the glutamatergic synapse [177], and its increased amount in the brain might further impairs ischemia-affected glutamatergic transmission. Furthermore, altered conditions of BCAAs and other neutral amino acids transported into the brain by the damaged blood–brain barrier might influence the balance of the transamination reactions, which play an important role in the intracerebral synthesis of glutamate and GABA, and might interfere with ammonia detoxification to glutamine in astrocytes [168].

According to the processes playing an important role in the etiopathogenesis of cerebral ischemia and stroke, such as excitotoxicity, neuroinflammation, and oxidative stress [14,178], Sidorov et al. [14] categorized blood metabolites altered in humans after a stroke into three groups. However, metabolites in the systemic circulation also have to fulfill other metabolic, energetical, and signaling functions in the particular body organs, which differs from their unique function in the CNS. For instance, glutamate plays a significant role in neuronal excitability, synaptic plasticity, immunity, and behavioral mechanisms such as learning and memory in the central nervous system [179]. However, it also serves as an important precursor or substrate for the synthesis of various amino acids, nucleic acids, nucleotides, and metabolites in other bodily tissues [180] as well as a regulator of the acid–base balance. Elevated blood glutamate levels found within one week after a stroke in one study [81] (Table 1) are unlikely to have originated directly from glutamate released post-ischemically by neurons in the brain, and this released glutamate in the brain, as discussed above, is also immediately utilized by glial cells. Therefore, the classification of amino acids altered after ischemia in the blood is ambiguous and has to be questioned. Alterations of each molecule in the plasma should be considered from the view of the complex mutual biochemical pathways in the comprehensive whole-body inter-organ metabolic exchange and communication.

**Table 1:** Alterations in liquid blood component (blood without cells) and extracellular microdialysate levels of amino acids found after cerebral ischemia in clinical and preclinical studies.

Metabolite					
Increase	Decrease	Study	Specimens	Analytical Technique	Reference
	glutamine, valine	patients after stroke < 72 h	liquid blood component	^1^H NMR	[80]
	histidine, isoleucine, leucine, methionine, proline, threonine	patients after stroke > 24 h	liquid blood component	MS	[181]
glutamine, proline, tyrosine	isoleucine, valine, tryptophan	post-stroke patients	liquid blood component	UHPLC/MS	[171]
aspartate	isoleucine, phenylalanine, proline, serine, valine	depressed post-stroke patients	liquid blood component	GC/MS	[182]
glutamate, tryptophan	alanine, leucine, isoleucine, methionine, proline, serine, tyrosine	post-stroke patients < 7 days	liquid blood component	GC/MS	[81]
	glycine, proline, serine, threonine, lysine, isoleucine, alanine	patients with acute stroke	liquid blood component	GC/MS	[183]
glutamate, cystine	proline	patients after stroke day 1	liquid blood component	ion exchange chromatography	[184]
glutamate, glycine, tyrosine, arginine, threonine, valine, leucine, phenylalanine	glutamine	patients after stroke day 5	liquid blood component	ion exchange chromatography	[184]
BCAAs, BCKAs	alanine, glutamine, tyrosine, lysine	rats 4VO, 3 h	liquid blood component	^1^H NMR	[84,86]
BCAAs, BCKAs, phenylalanine	alanine, glutamine, tyrosine, lysine	rats 4VO, 24 h	liquid blood component	^1^H NMR	[84,86]
BCAAs, phenylalanine, lysine		rats 4VO, 72 h	liquid blood component	^1^H NMR	[84,86]
lysine	tyrosine	rat 3 months, day 2 after MCAO	liquid blood component	^1^H NMR	[185]
lysine, isoleucine	tyrosine, proline, threonine	rat 12 months, day 2 after MCAO	liquid blood component	^1^H NMR	[185]
glutamate, aspartate, GABA		cats, global model of cerebral ischemia, up to 20 h	extracellular dialysate	microdialysis	[186]
benign group increased arginine, asparagine, BCAAs, methionine, phenylalanine, serine, and threonine against malignant group		patients with large middle cerebral artery infarction	extracellular dialysate	microdialysis	[17]

BCAAs—branched-chain amino acids, BCKAs—branched-chain ketoacids, GABA—gamma-aminobutyric acid, NMR—nuclear magnetic resonance, MS—mass spectroscopy, GC—gas chromatography, UHPLC—ultra-high-performance liquid chromatography, 4VO—four-vessel occlusion, MCAO—middle cerebral artery occlusion.

### 5.4. Issues Related to Metabolomic Studies of Cerebral Ischemia

The main post-ischemic changes of metabolites in the brain documented in experimental and clinical studies are, with a few exceptions, easily comparable. However, metabolic changes in amino acids found in plasma after cerebral ischemia do not always follow a unique pattern, and discrepancies can be found within clinical studies, and also between results from preclinical and clinical studies (Table 1). The comparison becomes challenging due to many issues, such as due to the following:

(i) Variability in sampling time after the ischemic event: Time interval from the onset of ischemia and the time range of the collection of samples. As was demonstrated in the preclinical studies by our group, the metabolic profile in plasma significantly changed over time [84,86].
(ii) In experimental studies, both the model selection, severity of ischemia, as well as the selection of suitable and relevant control groups affected the results. As we showed previously, differences in findings were observed in comparison to naïve animals and to sham-operated animals [84].
(iii) In human studies, stroke risk factors, such as diabetes, hypertension, dyslipidemia, and metabolic and vascular diseases, conferred distinct metabolic profiles; it is not known whether groups matched for such characteristics would retain implied significant metabolic distinctions, particularly given the limited number of participants, as a small number of observations (samples) produced a large number of variables (metabolites) [14].
(iv) The data presented from human studies are often a result obtained from a mixed population regardless of the gender of subjects. Although the generalized metabolic response to ischemia is assumed to be undifferentiated between females and males, it could partially differ when evaluating very low (a subtle) levels of metabolites in blood plasma [187]. One study presented gender-specific metabolic responses in the focal cerebral ischemia of rats in relation to plant extracts [188]. The fact is that experimental studies prefer to use only one gender, mainly male animals, to determine metabolic alterations; thus, the comparison between genders is not yet fully described. Although middle-aged women have a lower risk of stroke than middle-aged men, the menopausal transition is a time when many women develop cardiovascular risk factors. Additionally, during the 10 years after menopause, the risk of stroke roughly doubles in women [189]. During and after menopause, there may occur a significant change in body composition, and many women often gain weight (in the form of fat mass), which may further result in T2 diabetes mellitus development. All these facts surely highlight the need for gender-focused metabolomic phenotyping in relation to the metabolic response to cerebral ischemia and other disorders. Similarly, there is a lack of relevant metabolomic studies on cerebral ischemia focused on race and age comparisons.
(v) The functioning of the human body depends on the interaction of all organs, and injury to one can impact the others and produce compensatory/adaptation effects or secondary injuries. There is increasing evidence that the cerebral ischemic event is linked to further remote body organ/tissue damage. Post-ischemic vascular injury and BBB breakdown appear to be one of the most relevant events influencing the composition of the blood, ECF, or CSF levels of metabolites [28,190,191,192]; moreover, post-ischemic spleen atrophy, vascular and cardiac disorders [193], renal and pulmonary dysfunctions, liver injury, and alterations in pancreatic enzymes [194] are all conditions that might evolve after cerebral ischemia and therewith participate in alterations in the whole blood metabolome. Therefore, a critical and careful assessment of the observed metabolomic changes in plasma and brain extracellular fluid is necessary.
(vi) Practical issues: Inappropriate blood sampling procedures might increase blood lactate, or lactate might be increased by blood cells consuming glucose in the sampling tube if the blood is not processed within 2 h. Apart from this, particular metabolites are non-specifically bound to proteins. In NMR measurements in non-deproteinated plasma, they overtook the short transverse relaxation time T2 of the near protein and became invisible in cpmg-acquisition measurements [195]. The resulting signals originate only from ‘free’ metabolites. During the protein-removing procedure, the protein-bound metabolites are released, and the intensity of the signal arises; from there, the magnitude of the signal is dependent on the amount of protein that is present.

## 6. Analytical Platforms in Metabolomic Usable for Monitoring Cerebral Ischemia

Nowadays, metabolomic research is carried out using two basal instrumental platforms, mass spectrometry (MS) and NMR spectroscopy. NMR spectroscopy is characterized by a very high reproducibility [196] but lower sensitivity in comparison to MS, which shows high sensitivity and is balanced with average reproducibility. Both methods may work as supplements since they cover various spectra of metabolites. Apart from this, metabolomics research may include microdialysis and enzymatic methods combined with other types of spectroscopic or analytical methods, most frequently UV-vis or fluorescence spectroscopy.

A metabolomic investigation of the tissues may be performed non-invasively on living individuals using in vivo MRS, where metabolite levels are often expressed as ratios rather than absolute concentrations. However, the inter-subject variability of the denominator metabolite can introduce uncertainty into a metabolite ratio. Another limitation lies in the number of catchable metabolites, generally not more than ten, depending on the acquisition protocol. However, this method is the only one that offers opportunities for the non-invasive re-measurements of the same subjects at different time points. The spectra are the superposition of all signals from a voxel, regular grid in three-dimensional space, that should be precisely localized before the measurement in order to not catch signals from other structures.

Measurements of tissues in vitro are usually performed after homogenization and consecutive analysis, which leads to the results reflecting the whole tissue not being able to distinguish the contribution of intra- and extracellular space, and not being able to determine the localization of the most pronounced changes within the tissue or at a cell level. Apart from this, the removal of an organ from the body has to be performed very quickly, and metabolites with a very fast ischemic response cannot be responsibly evaluated.

Free unbound chemical substances from the extracellular medium in a specific location are often sampled with microdialysis. Substances may include endogenous molecules (neurotransmitters, glucose, etc.) or exogenous compounds; however, this method causes local trauma due to the implantation of the probe.

The monitoring of the ischemic response in the systemic circulation is much easier, where blood sampling can be performed repeatedly; however, the alterations in blood metabolites are the results of mutual biochemical pathways in the whole organism and may reflect effects from the injury as well as compensatory effects coming from the organs or following injury.

Together, the data obtained should always be assessed critically with a high focus on the limitation arising from the pre-analytical state (sample preparation) and the particular methodology. The most important advantages and limitations of NMR in comparison to MS spectroscopy in metabolomics applications were summarized by Emwas et al. [197]. For more details concerning the methodology, we also recommend the following reviews: [198,199,200,201].

## 7. Conclusions

A comprehensive evaluation of experimental and clinical studies suggests a very similar pattern of post-ischemic alterations in the levels of brain metabolites—lactate, glutamate, GABA, glutamine, and NAA—in affected brain parenchyma. Observed changes occur at various rates, from immediately released glutamate from neuronal cells and the production of lactate in the brain to relatively slowly—over days—evolving alterations in NAA as a putative manifestation of neuronal health or damage. The dual metabolic role of lactate in the reperfusion period and the very fast mutual conversion of glutamate/glutamine/GABA in the brain cannot be thought of as demonstrative correlators of the degree of damage and outcome both in the animal models as well as in human studies. The particular relation between metabolites and ischemic damage was demonstrated in preclinical studies, where animals which underwent the maneuver of ischemic preconditioning before ischemia generally showed decreased neuropathological damage; they also manifested a lower extent of metabolic and faster metabolic recovery after ischemia. Interestingly, hyperglycemia before ischemia could have a protective effect on ischemic injury when induced by glucose overdoses in animals; however, it is one of the main risk factors and is linked with aggravated outcomes after ischemic stroke when associated with diabetes type I or insulin deficiency in humans. This highlights the necessity to mimic and induce pathological conditions closer and more similar to real human pathology in their entire complexity in experimental models and in preclinical studies. The results of our experiments and other animal studies showed a switch in energy metabolism during post-ischemic reperfusion induced by both all common ischemic/reperfusion models, including MCAO or four-vessel occlusion, where glycolysis is partially balanced by the utilization of enhanced ketone bodies in the reperfusion period. 3-hydroxybutyrate is a major ketone body with a known neuroprotective effect; its production by this metabolic switch might be part of the body’s compensatory and/or self-adaptation process to adjust to post-ischemic conditions, mitigating neuronal damage. Summarized data from human stroke studies as well as the data from experimental animal studies suggest that the early normalization of the energy metabolism after ischemia is one of the key parameters in determining later cerebral post-ischemic damage and/or outcome. As observed in animal and human studies, stroke has a severe impact on peripheral organs; the alterations in amino acids in the blood circulation may be directly related to a turnover in energy, a protective and restorative metabolism environment after a cerebral ischemic event, and (from the perspective of the whole body) to an enhanced (i) compensatory immune response, (ii) preservation of the nitrogen shuttle among organs and acid–base balance, and (iii) important changes in the competition for transporting amino acids to the brain through the BBB. The proper transport of amino acids into the brain affects (i) the neurotransmitter’s precursors’ supply, (ii) the balance of the transamination and ammonia detoxification, as well as (iii) the ability to restore neurotransmission after ischemia. It is now widely accepted that due to the interaction of all bodily organs, an injury to one organ influences the others and initiates compensatory effects or cause secondary injuries. In humans, post-ischemic conditions are often accompanied by an infringement in cardiac, pulmonary, renal, and other tissue functions, which is reflected by the alterations that occur in relation to the general blood metabolome. Therefore, a critical and careful assessment of the post-ischemic changes in blood metabolites and substantial, regulated, and focused metabolic support in human acute and chronic cerebrovascular pathologies is urgently required.
